# The Prevalence of Non-infectious Diseases Among Overseas Chinese Workers in 2018

**DOI:** 10.3389/fepid.2022.817850

**Published:** 2022-07-19

**Authors:** Bolin Zhang, Xiangguang Ye, Qi Chen, Qinqin Jiang, Xueying Zhang, Lian Tong

**Affiliations:** ^1^Hefei Preschool Teachers College, Hefei, China; ^2^Anhui Import and Export Inspection and Quarantine Bureau, Hefei, China; ^3^Hefei Preschool Education College, Hefei, China; ^4^Department of Epidemiology, Human Gneetics and Environmental Sciences, School of Public Health, The University of Texas Health Science Center at Houston, Houston, TX, United States; ^5^Department of Maternal, Child and Adolescent Health, Key Laboratory Public Health Safety, Fudan University, Shanghai, China

**Keywords:** hypertension, migrant worker, non-infectious disease, disease surveillance, global health

## Abstract

**Background:**

With the “Belt and Road” initiative, more Chinese citizens have gone abroad to engage in overseas labor activities. Few studies have investigated the prevalence of non-infectious diseases among Chinese overseas workers. This study seeks to fill the gap and illustrate the relevant diseases in a population of Chinese overseas workers.

**Methods:**

The health records of 13,529 Chinese migrant workers (12,917 males, mean age 41.3 ± 8.7 years, and 612 females, mean age 33.1 ± 10.2 years) who visited the International Travel Health Care Center in Anhui province were obtained. Chi-square test and binary logistic regression models were used to analyze the associations between the prevalence of non-infectious diseases and sex, as well as the association between non-infectious diseases and length of stay abroad.

**Results:**

In this study, 34.6% of overseas workers were found to have one or more types of non-infectious diseases. Hypertension had the highest prevalence (9.58%). Hypertension, fatty liver, renal disease and abnormal liver function tests were more prevalent among male workers than among female workers, while anemia and abnormal urinalysis were more prevalent among female workers. The prevalence of hypertension, renal diseases, liver diseases and gallbladder diseases increased with the length of stay abroad.

**Conclusion:**

Non-infectious diseases including cardiovascular diseases, diabetes, and chronic respiratory diseases were highly prevalent among Chinese overseas workers. Hence the monitoring of non-infectious diseases needs to be enhanced to reduce China's overall disease burden in the future.

## Introduction

With the “Belt and Road” initiative, an increasing number of Chinese citizens have gone abroad to engage in overseas labor activities. China is one of the largest export countries of migrant workers. According to China's Ministry of Commerce, a total of 1.027 million Chinese citizens worked abroad during the year of 2015, an increase of 21,000 from 2014 (1). China's overseas workers mainly are rural population, who have relatively low education level, mainly engaged in relatively low-technology-intensive work such as construction, transportation and manufacturing (2). Nearly half of these Chinese overseas workers worked in areas disadvantaged in public health, for example, in countries in Africa with a high prevalence of poverty, AIDS, hepatitis, and other public health problems (3). In addition to the disadvantaged public health situations in these countries, the changes in their living environment and diet, as well as work-related and environmental stressors increased the risk of developing chronic health conditions among migrant workers (4).

The Chinese government has implemented several health policies and interventions to prevent Chinese overseas workers from developing adverse health outcomes. For example, the monitoring of infectious diseases in Chinese overseas workers has been implemented in China for many years. However, there is no established monitoring system for non-infectious diseases. One study of US travelers found that 49% of all deaths were due to cardiovascular events, much more than deaths due to accidents and infectious causes combined. Others have described unique challenges to chronic disease management associated with travel (5).

Non-infectious diseases account for 70% of total mortality on a global scale (6). For the working population, non-infectious diseases are a considerable risk factor for loss of work time, and contribute to enormous health care expenses (7). The prevention of non-infectious diseases requires knowledge about distribution and risk factors, including the profiles of people at high risk of developing them. Therefore, our study aims to investigate the prevalence and risk factors for non-infectious diseases in Chinese migrant workers working abroad. The study results will become a reference to designing preventive strategies in our study population, who generally have low socio-economic status and lack of access to high-quality healthcare services (8).

## Methods

### Data Source

Our study population is a group of Chinese workers who are working overseas. Most of them are from Anhui province. The medical records and physical examination reports of all Chinese overseas workers who visited the International Travel Health Care Center in Anhui province during January 1st to December 31st, 2018 were obtained. According to China's policy, all workers who are expected to stay abroad for more than 1 year or have already stayed abroad for more than 3 months are required to have a physical examination in the respective International Travel Health Care Centers in their home province. Thus, our data covered almost all of the overseas workers who were identified as permanent residents under employment in Anhui province in the year of 2018.

### Data Collection

All of the migrant workers were asked to complete a questionnaire during their visits. The questionnaire included demographic information (e.g., age, sex, education level, country of work, travel history in the past 5 years, and self-reported disease history). Physical examination was conducted for each overseas worker at every clinic visit, included metric body measurement, blood test, spot urine test, liver and renal function tests, electrocardiography, chest X-ray, and abdominal ultrasound. The following reference ranges were used to determine normal results systolic blood pressure <140 mmHg, diastolic blood pressure <90 mmHg; alanine aminotransferase (ALT) 0–40. Electrocardiographs (ECG) were interpreted, with common abnormalities being sinus tachycardia, sinus bradycardia, premature beats, T wave abnormalities, ST segment abnormalities, etc. Ultrasound of the kidneys and hepatobiliary system were performed, with common abnormalities including fatty liver, hepatic cysts, gallbladder polyps, hepatic hemangioma, features of early cirrhosis, renal cysts, renal calculus, renal hydronephrosis, etc. The normal reference range of full blood count are as follows, white blood cell count (4.5–10.0) x 10^9^ / L, red blood cell count: male (4–5.5) x 10^12^ / L, female (3.5–5.0) x 10^12^ / L, hemoglobin quantification: 120–160g/ L for males, 110–150 g / L for females, platelet count (100–300) x 10^9^ / L.

Definition of grouping: (1) Kidney diseases: including kidney stones, renal cysts and hydronephrosis; (2) Liver diseases: including early cirrhosis, hepatic cysts and intrahepatic hemangioma; (3) Gallbladder diseases: including gallbladder polyps and gallstones; (4) Urinary glucose abnormalities: urinary glucose positive and +++ above; (5) Coagulation abnormalities: Abnormal platelet count.

### Statistical Method

Counts (and percentages) of categorical variables and means (SDs) of continuous variables were calculated respectively for demographic characteristics and non-infectious diseases. Chi-square test was used to examine the differences in disease prevalence across genders. A binary logistic regression model was used to examine the age-adjusted associations between individual type of non-infectious diseases and gender, as well as the age-adjusted and gender-adjusted association between individual type of non-infectious diseases and length of stay abroad. In the binary logistic regression, the study population was further divided into three categories according to the length of stay abroad. The three categories were those (a) who had never worked abroad, (b) those who had worked abroad between 1 and 2 years, and (c) those who had worked abroad for more than 2 years. A two-tailed *p*-value < 0.05 was considered as statistically significant. Data analyses were performed using SPSS16.

### Ethical Consideration

The analysis of this data was exempted by the local IRB of Medical ethics committee of Fudan University (IRB#2019-11-0787). Oral informed consent was obtained from each participant before the physical examination and specimen collection after a thorough explanation of the study's purposes and risks. All data were analyzed anonymously, and any patient identifier was removed to maintain patient confidentiality.

## Results

### Demographic Characteristics

A total of 13,529 Chinese overseas workers completed both the questionnaire and physical examination in the International Travel Health Care Center in Anhui province in 2018. All of the workers were Chinese citizens, 12,917 participants were male (mean age 41.3 ± 8.7 years), and 612 participants were female (mean age 33.1 ± 10.2 years). Overall, there was a obvious preponderance of male travelers. Most people spend <2 years working aboard. The participants were reported to be working in 150 countries and administrative regions. The top 10 places ranked by the number of overseas Chinese workers in descending order were Algeria, Angola, Saudi Arabia, Malaysia, Panama, Macao, Ethiopia, Mozambique, Zambia, and Japan. Among all continents, Africa had the largest number of overseas Chinese workers (8,609, 63.63%), followed by Asia, South America, Europe, North America, and Oceania (Table 1).

**Table 1:** Demographic characteristics among Chinese migrant workers.

**Demographic characteristics**	**Population (%)**
**Age**	
18–30	2,244 (16.6)
31–50	9,607 (71.0)
>50	1,678 (12.4)
**Sex**	
Male	12,917 (95.5)
Female	612 (4.5)
**Number of years abroad**	
<1	8,823 (65.2)
1–2	2,810 (20.8)
≥2	1,896(14.0)
**By countries (top 10)**	
Algeria	3,873 (28.6)
Angola	1,550 (11.5)
Saudi Arabia	655 (4.8)
Malaysia	565 (4.2)
Panama	402 (3.0)
Macau, S.A.R.	400 (3.0)
Ethiopia	391 (2.9)
Mozambique	316 (2.3)
Zambia	285 (2.1)
Japan	277 (2.1)
**By continents**	
Africa	8,609 (63.6)
Asia	3,640 (26.9)
South America	652 (4.8)
Europe	396 (2.9)
North America	148 (1.1)
Oceania	84 (0.6)

### Distribution of the Non-infectious Diseases in Chinese Overseas Workers

Among the 13,529 investigated migrant workers, 4,683 (34.6%) had one or more types of non-infectious diseases, and 165 (1.2%) had more than three types. Male workers have a higher prevalence of non-infectious diseases (chi-square *P*-value < 0.0001) than female workers (Table 2).

**Table 2:** The numbers of non-infectious diseases among Chinese migrant workers, listed by gender.

**Number of non-infectious diseases**	**Gender (%)**		**Total**
	**Male**	**Female**	
0	8,370 (64.8)	476 (77.8)	8,846 (65.4)
1	3,521 (27.3)	111 (18.1)	3,632 (26.8)
2	865 (6.7)	21 (3.4)	886 (6.6)
3	153 (1.2)	3 (<0.01)	156 (1.2)
4	8 (<0.01)	0 (0)	8 (<0.01)
5	0 (0)	1 (<0.01)	1 (<0.01)
Total	12917 (95.5)	612 (4.5)	13,529(100)
*P*-value^*^	<0.0001

* *The p-value is calculated from chi-square test. The two factors in the comparison were gender and disease status (yes/no)*.

### Gender Difference

Among all of the investigated non-infectious diseases, the most prevalent disease in our study population is hypertension (1,296, 9.58%). According to the results of logistic regression, individual type of the non-infectious diseases shows varied gender distribution. Female workers have lower odds of hypertension (age-adjusted OR: 0.52, 95% CI: 0.34, 0.82), fatty liver (age-adjusted OR: 0.39, 95% CI: 0.19, 0.84), elevated alanine aminotransferase (age-adjusted OR: 0.30, 95% CI: 0.122, 0.737) and renal dysfunction (age-adjusted OR: 0.59, 95% CI: 0.35, 0.99) than male workers. Male workers have lower odds of anemia (age-adjusted OR: 63.73, 95% CI: 10.86, 362.45) and abnormal urinalysis (age-adjusted OR: 15.78, 95% CI: 7.02, 35.45) than female workers (Table 3).

**Table 3:** Distribution of non-infectious disease among Chinese migrant workers who were working abroad.

**Non-infectious disease**	**Male**	**Female**	**Total**	Adjusted OR^*^(95%CI)
	***N* (%)**	***N* %)**	***N* (%)**	
Hypertension	1,275 (9.87)	21 (3.43)	1,296 (9.58)	0.52 (0.34, 0.82)
Pulmonary disease	23 (0.18)	0 (0)	23 (0.17)	NA^**^
Abnormal urine glucose (>3+)	57 (0.44)	1 (0.17)	58 (0.43)	0.56 (0.08, 4.05)
Anemia	2 (0.02)	4 (0.65)	6 (0.04)	62.73 (10.86, 362.45)
Elevated alanine aminotransferase (ALT>40)	248(1.92)	5(0.04)	253(1.87)	0.30(0.122, 0.737)
Fatty liver	363 (2.81)	7 (1.14)	370 (2.73)	0.39 (0.19, 0.84)
Renal dysfunction	734 (5.68)	15 (2.45)	749 (5.54)	0.59 (0.35, 0.99)
Liver diseases	555 (4.30)	15 (2.45)	570 (4.21)	0.96 (0.57, 1.63)
Gallbladder disease	671 (5.19)	29 (4.73)	700 (5.17)	1.19 (0.81, 1.75)
ECG abnormalities	407 (3.15)	16 (2.61)	423 (3.13)	1.12 (0.67, 1.87)
Coagulation abnormalities	62 (0.48)	3 (0.49)	65 (0.48)	1.33 (0.41, 4.32)
Abnormal urinalysis	18 (0.14)	11 (1.80)	29 (0.21)	15.78 (7.02, 35.45)
Total investigated population	12,917	612	13,529	

* *Adjusted ORs were calculated from logistic regression, considered age as a confounder and male as a reference group*.

** *No female worker was found to have pulmonary disease*.

### Correlation Between Abnormal Test Results and Length of Work Overseas

Table 4 shows the results of binary logistic regression concerning the odds of non-infectious diseases and length of stay abroad. In the results (Table 4), the odds of hypertension (age-adjusted OR: 1.07, 95% CI: 1.01, 1.85), renal diseases (age-adjusted OR: 1.11, 95% CI: 1.01, 1.23), hepatic diseases (age-adjusted OR: 1.14, 95% CI: 1.02, 1.27), and gallbladder diseases (age-adjusted OR: 1.20, 95% CI: 1.03, 1.41) statistically significantly increased as length of stay abroad increased.

**Table 4:** The association between length of work abroad and prevalence of non-infectious diseases.

**Health status**	**Number of years working abroad**			**Adjusted OR** (95% CI)^*^
	** <1 [*N* (%)]**	**1–2 [*N* (%)]**	**≥2 [*N* (%)]**	
Hypertension				1.07 (1.01,1.85)
No	8,006 (90.7)	2,531 (90.1)	1,696 (89.5)	
Yes	817 (9.3)	279 (9.9)	200 (10.5)	
Coagulation abnormalities				1.28 (0.94,1.73)
No	8,786 (99.6)	2,797 (99.5)	1,881 (99.2)	
Yes	37 (0.4)	13 (0.5)	15 (0.8)	
Elevated alanine aminotransferase (ALT>40)				1.16(0.98,1.38)
No	8,661 (98.2)	2,759 (98.2)	1,856 (97.9)	
Yes	162 (1.8)	51 (1.8)	40 (2.1)	
Abnormal urine glucose (>3+)				0.85 (0.59,1.23)
No	8,784 (99.6)	2,798 (99.6)	1,889 (99.6)	
Yes	39 (0.4)	12 (0.4)	7 (0.4)	
Fatty liver				0.96 (0.83,1.11)
No	8,577 (97.2)	2,735 (97.3)	1,847 (97.4)	
Yes	246 (2.8)	75 (2.7)	49 (2.6)	
Renal dysfunction				1.11 (1.01,1.23)
No	8,380 (95.0)	2,636 (93.8)	1,764 (93.6)	
Yes	443 (5.0)	174 (6.2)	132 (7.0)	
Liver diseases				1.14 (1.02,1.27)
No	8,492 (96.2)	2,683 (95.5)	1,784 (94.1)	
Yes	331 (3.8)	127 (4.5)	112 (5.9)	
Gallbladder disease				1.20 (1.03,1.41)
No	8,409 (95.3)	2,626 (93.5)	1,794 (94.6)	
Yes	414 (4.7)	184 (6.5)	102 (5.4)	
ECG abnormalities				0.97 (0.85,1.11)
No	8,551 (96.9)	2,722 (96.9)	1,833 (96.7)	
Yes	272 (3.1)	88 (3.1)	63 (3.3)	
Abnormal urinalysis				0.48 (0.23,1.00)
No	8,799 (99.7)	2,806 (99.9)	1,895 (99.9)	
Yes	24 (0.3)	4 (0.1)	1 (0.1)	

* *Adjusted ORs were calculated from logistic regression, considered age and gender as a confounder*.

## Discussion

Based on our results, non-infectious diseases were a considerable health threat among overseas Chinese workers. Among the non-infectious diseases investigated, hypertension was the most prevalent in our study population, but still lower than the Chinese national average, probably because of The Healthy Workers Effect (9). Male workers had a higher overall prevalence of non-infectious diseases than females. Several types of diseases show significant gender difference. Hypertension, elevated alanine aminotransferases (ALT > 40), fatty liver, and renal disease were more common in male workers than in female workers; anemia and urinalysis abnormalities were more common in female workers than male workers. In addition, length of stay abroad significantly increased the odds of hypertension, renal diseases, hepatic diseases, and gallbladder diseases.

Hypertension was highly prevalent in our study population (10). This findings of our study are consistent with the findings of other studies focused on migrant workers in China. For example, a study of migrant Chinese workers within the country found that the migrant population had higher blood pressures compared to their urban neighbors and residents in their home villages (11).

In this study, male workers were found to have a higher prevalence of all types of non-infectious diseases. However, female workers were found to have a statistically significant higher prevalence of anemia or abnormalities in urinalysis than those of male workers. This could be related to the physiological differences, as women are more prone to genitourinary diseases and iron-deficiency anemia than men (12). Among our study participants, male workers have a higher prevalence of hypertension, raised ALT, fatty liver, and renal disease than women, which could be related to a higher rates of smoking and alcohol use in Chinese men (13). However, poor working conditions overseas, such as in Africa, language barriers and homesickness may increase the risk.

The economic development in African countries in recent years has provided more opportunities for Chinese workers overseas, however, the relatively under-developed health care system can pose considerable healthcare challenges on these overseas workers. In addition, Chinese workers in Africa typically had longer working contracts compared to those in other countries (14). A certain proportion of workers also had preexisting hypertension, cardiac conditions, diabetes or other chronic diseases before leaving for other countries. The transition in living environment and work stress could play an essential role in causing the high incidence of non-infectious diseases among overseas Chinese workers. Finally, there is a lack of occupational health monitoring systems particularly focused on disease screening and management in this population (15). The majority of those overseas workers experience long work duration, frequent contact with hazardous chemical components, and a scarcity of fresh food. Most of them had less than high school education level and limited knowledge about disease prevention (4), as well as difficulties in accessing healthcare due to language barriers. Those factors also contribute to the development of non-infectious diseases among Chinese overseas workers.

The length of stay abroad is another risk factor for non-infectious diseases. Chinese overseas workers who spent more years overseas had higher incidences of hypertension, renal dysfunction, Liver diseases, and gallbladder diseases. This could be related to problems with acclimatization, dietary changes, occupational stress, poor living conditions, unhealthy living habits (16). Most Chinese overseas workers working abroad are young individuals who engage in labor-intensive work, have limited knowledge of healthcare, and are less health conscious when it comes to diet. This may have contributed to the insidious development of cardiovascular and gastrointestinal tract diseases in young, healthy workers. In addition language barriers problem also made it difficult for workers to adapt to the new environment, In the face of this situation, the occupational health agency in Chinese government should consider providing long-term guidance and follow-ups for Chinese nationals working abroad (17).

Previous research has shown that cardiovascular diseases are the leading cause of death among migrant workers (18–21). Many types of cardiovascular diseases are highly prevalent in our study population. Currently, we have no statistics regarding the mortality in these workers (22). However, the monitoring of cardiovascular diseases in Chinese migrant workers is still important for disease prevention and disease burden reduction. To monitor the health status of overseas workers in their working countries is difficult, due to logistics and policy consideration. Hence China's governmental agencies at the provincial level need to pay closer attention to the existing health conditions of migrant workers. For example, low-cost treatment, affordable health insurance, and free medications are feasible approaches in China's province-level overseas worker clinics.

## Conclusions

The prevalence of non-infectious diseases in Chinese overseas workers has not garnered much attention. Based on our data certain types of non-infectious diseases (e.g., hypertension) are highly prevalent in Chinese overseas workers. Non-infectious diseases pose a severe life threat to these overseas workers, and to them is difficult due to logistics and political issues. Therefore, the control and prevention of non-infectious diseases in their home country is crucial. Although a pre-travel health consultation cannot guarantee a traveler's health and safety, a well-organized and well-executed consultation using counseling, education and administration of vaccines and prescriptions can help reduce and manage the risks of illness and injury during travel. Governmental agencies like the Anhui Import and Export Inspection and Quarantine Bureau can pay more attention to the health status of overseas workers, including providing affordable healthcare and healthy lifestyles guidance. Our study can help to guide the design of specific strategies in controlling and preventing non-infectious diseases among Chinese overseas workers.

## Data Availability Statement

The original contributions presented in the study are included in the article/supplementary material, further inquiries can be directed to the corresponding author.

## Ethics Statement

The analysis of this data was exempted by the local IRB of Medical Ethics Committee of Fudan University (IRB#2019-11-0787). The patients/participants provided their written informed consent to participate in this study.

## Author Contributions

All authors listed have made a substantial, direct, and intellectual contribution to the work and approved it for publication.

## Funding

This study was supported by the China Anhui University Natural Science Foundation-Funded Project 2021 (KJ2021A1303), the Anhui Provincial Level Quality Project Teaching Research General Project 2021 (2021gkszgg059), and the Key Discipline Program of the Fifth Round of the Three-Year Public Health Action Plan (2020-2022) of Shanghai (GWV-10.1-XK08).

## Conflict of Interest

XY was employed by Anhui Import and Export Inspection and Quarantine Bureau. The remaining authors declare that the research was conducted in the absence of any commercial or financial relationships that could be construed as a potential conflict of interest.

## Publisher's Note

All claims expressed in this article are solely those of the authors and do not necessarily represent those of their affiliated organizations, or those of the publisher, the editors and the reviewers. Any product that may be evaluated in this article, or claim that may be made by its manufacturer, is not guaranteed or endorsed by the publisher.
